# Acute respiratory distress syndrome: how do patients fare after the intensive care unit?

**DOI:** 10.5935/0103-507X.20190074

**Published:** 2019

**Authors:** Roselaine Pinheiro de Oliveira, Cassiano Teixeira, Régis Goulart Rosa

**Affiliations:** 1 Unidade de Terapia Intensiva, Hospital Moinhos de Vento - Porto Alegre (RS), Brasil.; 2 Departamento de Clínica Médica, Universidade Federal de Ciências da Saúde de Porto Alegre - Porto Alegre (RS), Brasil.; 3 Programa de Pós-Graduação em Ciências da Reabilitação, Universidade Federal de Ciências da Saúde de Porto Alegre - Porto Alegre (RS), Brasil.; 4 Unidade de Terapia Intensiva, Hospital São Lucas, Pontifícia Universidade Católica do Rio Grande do Sul - Porto Alegre (RS), Brasil.; 5 Unidade de Terapia Intensiva, Hospital de Clínicas de Porto Alegre, Universidade Federal do Rio Grande do Sul - Porto Alegre (RS), Brasil.

**Keywords:** Respiratory distress syndrome, adult, Quality of life, Intensive care units

## Abstract

Patients with acute respiratory distress syndrome require ventilation strategies that have been shown to be important for reducing short-term mortality, such as protective ventilation and prone position ventilation. However, patients who survive have a prolonged stay in both the intensive care unit and the hospital, and they experience a reduction in overall satisfaction with life (independence, acceptance and positive outlook) as well as decreased mental health (including anxiety, depression and posttraumatic stress disorder symptoms), physical health (impaired physical state and activities of daily living; fatigue and muscle weakness), social health and the ability to participate in social activities (including relationships with friends and family, hobbies and social gatherings).

## INTRODUCTION

Acute respiratory distress syndrome (ARDS) is characterized by bilateral opacities, noncardiogenic pulmonary edema and hypoxemia, with arterial oxygen partial pressure to fractional inspired oxygen ratio (PaO_2_/FIO_2_) < 300 and positive end-expiratory pressure (PEEP) ≥ 5cmH_2_O; it may occur in response to different insults, such as sepsis, trauma, pneumonia or massive transfusion.^(1,2)^ Due to hypoxemia, patients with ARDS require ventilation strategies (protective ventilation and prone ventilation) that have been shown to be important in reducing short-term mortality.^(3-5)^ However, surviving patients have prolonged stays in both the intensive care unit (ICU) and the hospital, and they experience reduced overall satisfaction with life (independence, acceptance and positive outlook), mental health (anxiety, depression and symptoms of posttraumatic stress disorder - PTSD), physical health (physical state, activities of daily living, fatigue and muscle weakness), social health and the ability to participate in social activities (relationships with friends and family, hobbies and social gatherings).^(6)^ This combination of sequelae is called "post-intensive care syndrome" and is a complex combination of cognitive, psychological and motor symptoms.^(7)^

The objective of this review was to describe findings of post-intensive care syndrome in patients with ARDS who survived the ICU stay.

## LONG-TERM MORTALITY

Unlike other critical diseases, ARDS is associated with a substantial risk of in-hospital mortality but a surprisingly low risk of long-term mortality. Apparently, the first six months after discharge represent the period of greatest lethality for this population, and the estimated mortality of ARDS survivors is 12% at 1 year, 15% at 2 years and 19% at the 5-year follow-up.^(8)^ These data differ from the findings of general cohorts of ICU survivors, for whom excessive mortality in the first 5 years of follow-up is reported. These cohorts include patients with acute exacerbation of chronic obstructive pulmonary disease (COPD) or with respiratory failure secondary to septic shock, clinical situations with pathophysiological mechanisms of lung injury different from those found in ARDS. In particular, patients with COPD tend to have poor long-term outcomes after severe illness, with a 5-year mortality rate of 76%.^(8)^

## PHYSICAL STATE AND ACTIVITIES OF DAILY LIVING

Biehl et al.^(9)^ evaluated the functional status (using the 12-Item Health Survey - SF-12 and Barthel's index) of ARDS survivors six months after discharge from the ICU and found no differences when compared to critically ill patients without ARDS. The inability to perform activities of daily living prior to ICU admission seems to be an important marker of functional decline in this population.^(9)^ In turn, the severity of respiratory failure does not seem to affect prognosis according to a comparison of patients who underwent extracorporeal membrane oxygenation (ECMO) with those who did not.^(10)^

The impact of post-ICU muscle weakness was assessed in 156 survivors of ARDS.^(11)^ At hospital discharge, 38% of the survivors were diagnosed with muscle weakness, and the 5-year mortality rate was three times higher in this group. Each point on the Medical Research Council (MRC) scale was associated with increased survival (hazard ratio (HR): 0.96; 95% confidence interval (95%CI): 0.94 - 0.98), and after 5 years, 50% of the survivors still had muscle weakness. Interestingly, even those who recovered muscle strength after hospital discharge experienced significantly high mortality. Patients with ICU-acquired weakness have significant morphological muscle damage.^(11,12)^ Studies that included muscle biopsies of ARDS survivors showed chronic myopathic changes up to 2 years after the acute episode, suggesting that the residual muscle injury may be correlated with the functional disability observed in these patients.^(13-15)^ In addition, there is a significant loss of lean mass in some of these patients.^(16)^

Brosky et al.^(17)^ demonstrated that one-third of survivors of ARDS have symptoms of dysphagia at the time of hospital discharge but experience full recovery of symptoms after 5 years of follow-up.

## MENTAL STATE

The psychiatric dysfunction of post-ICU syndrome involves anxiety, depression and PTSD. Symptoms of PTSD are found less frequently than symptoms of anxiety and depression, regardless of the length of follow-up;^(18,19)^ however, the prevalence of PTSD does not differ from that of populations of critically ill patients without ARDS.^(20)^ At hospital discharge, approximately 40% of patients with ARDS present with PTSD.^(21)^ At one year of follow-up, symptoms of anxiety and depression occur in 66% of cases (416/629 patients).^(20)^ Two years after discharge from the ICU, the prevalence of PTSD is 22% to 24%,^(22)^ that of anxiety is 38% to 44% and that of depression is 26% to 33%.^(23)^ After 5 years, 28% had a diagnosis of PTSD, and after 8 years, 23.9% did.^(21)^ Interestingly, many patients exhibit symptoms in all three psychiatric domains simultaneously.

Similar to findings regarding the physical state, greater severity of ARDS does not seem to correlate with the prevalence of psychiatric symptoms after discharge from the ICU.^(21,24)^ However, younger age, unemployment, female gender and alcohol use were related to a higher prevalence of psychological syndromes. In these subgroups, clinically significant persistent or recurrent symptoms of anxiety (38%), depression (32%) and PTSD (23%) were common in the first 5 years after ARDS.^(24)^

The etiology of psychiatric disorders associated with ARDS is unknown.^(21)^ Most of the literature suggests that pathophysiological changes related to critical illness (hypoxemia, activation of the hypothalamic-pituitary axis, elevated cytokines and organ dysfunction) and to the use of drugs (norepinephrine and sedatives) contribute to the onset of long-term psychological disorders.^(24)^ A previous history of depression is strongly associated with psychiatric morbidity after ARDS.^(24)^

The social impact of depression is substantial because patients with moderate to severe psychiatric symptoms have greater difficulty returning to work than those with mild to moderate symptoms.^(25)^ Individuals with PTSD have a greater tendency toward somatization and anxiety in addition to major impairment in some dimensions of health-related quality of life (HRQoL), such as general health, social function and mental health.^(26)^ A positive correlation was found between the number of traumatic memories and the experience of anxiety and the severity of PTSD.^(26)^ Regarding aspects related to ICU interventions, the duration of sedation and mechanical ventilation are considered long-term predictors of PTSD.^(27)^

## COGNITIVE STATE

Little is known about the pathophysiology of neurocognitive impairment after ARDS. It is likely that different mechanisms contribute to the development of neurocognitive dysfunction (hypoxemia, delirium, changes in blood glucose, the effects of sedatives and pre-existing cognitive impairment). Approximately 50% of survivors may develop cognitive dysfunction in the long term (1 to 2 years), especially in terms of attention, memory, mental processing speed and executive function.^(9)^ Biehl et al.^(9)^ found no difference in the SF-12 mental component between critically ill patients with and without ARDS at a long-term evaluation. The classic study by Pandharipande et al.^(28)^ found that critically ill patients with shock or acute respiratory failure had a high risk of cognitive impairment in the first year after hospital discharge. In addition, one-fourth of elderly patients (> 65 years) had neurological examination results compatible with dementia after 1 year of follow-up. In a cross-sectional study,^(24)^ at the 12^th^ month after ICU discharge, 71% of the patients had abnormal neuropsychological tests. Additionally, in this domain, the severity of the disease does not seem to increase the risk of cognitive deficit, based on the comparison of patients with ARDS who did and did not receive ECMO.^(10)^

## PULMONARY FUNCTION

In patients with ARDS, the reduction in pulmonary function does not seem to be as significant as initially thought.^(12,13)^ Spirometry (which assesses static and dynamic pulmonary volumes) and diffusion capacity (which assesses the capacity for gas exchange through the alveolar barrier) are the techniques used to assess pulmonary function.^(29)^ The 6-minute walk test (which assesses overall cardiopulmonary function) is also used to assess pulmonary function alone.^(29)^ Of these, diffusion capacity seems to be the parameter that is universally acutely affected, and although there was an improvement from 62 - 63% to 72 - 77% of the predicted value, it remained at the lower limit of normal during the first year after ARDS.^(30.31)^ Studies indicate that lung volumes show a strong tendency to return to normal 3 to 6 months after the acute phase.^(12,13,30,31)^ However, 6% to 43% of patients develop an obstructive pattern and 15% to 58% develop a restrictive pattern in the first year of follow-up.^(12,13,30,31)^ It is believed that a restrictive pattern may be explained by the progression to pulmonary fibrosis and/or the development of ICU-acquired respiratory muscle weakness. One year after discharge, patients had increase in the distance covered in the 6-minute walk test compared to the findings in the immediate postdischarge period.^(30,31)^ There is still controversy regarding whether the inability to exercise is due to dyspnea or muscle weakness. It is likely to be multifactorial, as no correlation was found between parenchymal abnormalities detected with computed tomography, respiratory symptoms, pulmonary function tests and the 6-minute walk test. It is important to note that some patients maintain the changes in diffusion capacity and the 6-minute walk test for up to 5 years after ARDS.^(21)^

Although short-term survival is significantly better in patients with ARDS who are ventilated using a protective strategy, there is no evidence of altered pulmonary function up to two years after the resolution of the acute phase compared to conventionally ventilated patients.^(30)^ There also appears to be no difference in spirometric values between patients with pulmonary and extrapulmonary ARDS or between those ventilated in the prone position and those ventilated in the standard position.^(30)^

## RETURN TO WORK AND REHOSPITALIZATION

The possibility of returning to work is an important quality of life indicator for critical illness survivors. Myhren et al.^(32)^ showed that 55% of previously active patients with ARDS returned to work or school within 1 year of follow-up. A recent study^(33)^ conducted with 922 ARDS survivors in 43 American hospitals showed that 44% of patients were unemployed 1 year after hospital discharge. A reduction in earnings was reported by 71% of patients, and the variables associated with unemployment were length of hospital stay and age.

Ruhl et al.^(34)^ found that 40% of ARDS survivors experience at least one hospitalization after discharge during the first 12 months of follow-up. Physical or psychological decline was associated with subsequent hospitalization.

## HEALTH-RELATED QUALITY OF LIFE

There are still many uncertainties in the assessment of HRQoL in patients with ARDS after their discharge from the ICU.^(35)^ Differences in assessment time points and the heterogeneity of the scales used complicate the interpretation of findings.^(36)^

In a meta-analysis,^(35)^ the HRQoL of ARDS survivors was significantly decreased in the first year after ICU discharge compared to that of the general population, especially due to worsening of the physical domain. In a secondary analysis of the OSCAR study (n = 795),^(37)^ ARDS survivors reported a significantly lower HRQoL than the age- and sex-matched reference population. This finding was more marked in patients younger than 65 years. Bienvenu et al.^(22)^ demonstrated that patients had at least one psychological diagnosis associated with a reduction in the physical domain of HRQoL at 2 years after hospital discharge. In a multivariate analysis, improvement in physical performance was associated with a greater likelihood of remission of psychiatric symptoms. In an assessment of the HRQoL of patients five years after ICU discharge, ARDS survivors had a 25% reduction in physical function and a 17.5% reduction in general health compared to the general population.^(38)^ Even younger individuals (< 52 years old)^(38)^ and individuals with few comorbidities^(39,40)^ showed a reduction in the physical domain.

Additionally, in an assessment of HRQoL, the severity of ARDS did not seem to influence long-term outcomes.^(10)^ In that study, ECMO and non-ECMO survivors had similar HRQoL one year after ICU discharge, but when compared with the general population, both groups showed significantly lower HRQoL.

Curiously, ARDS survivors seem to display four distinct long-term progression patterns: (a) mildly impaired physical and mental health, (b) moderately impaired physical and mental health, (c) severely impaired physical health with moderately impaired mental health and (d) severely impaired physical and mental health.^(41,42)^ This phenotypic division may, in the near future, guide therapy after hospital discharge.

To clarify the specific contribution of ARDS to long-term outcomes, some studies^(14,43)^ compared the HRQoL of ARDS survivors with that of ICU survivors without ARDS and found no difference at six months after discharge. However, these results should be interpreted with caution considering the heterogenous criteria for acute lung disease included in the definition of ARDS over the years and the different baseline characteristics of studied populations in terms of age, preexisting lung disease and number of comorbidities.

## PREVENTION OF SEQUELAE

Little is known about the prevention of post-intensive care syndrome.^(7,8)^ Early mobilization therapy has been reported to prevent or attenuate the impairment of physical function in critically ill patients.^(44)^ An improvement in physical function associated with early goal-oriented mobilization was observed in a cohort of surgical patients;^(45)^ however, these positive results have not yet been replicated in patients with acute respiratory failure.^(46,47)^ The support of family and caregivers during the ICU stay and motor rehabilitation seem to prevent the onset of PTSD.^(26,48)^

## FINAL COMMENTS

Although mortality from ARDS has decreased in recent years, those who survive develop changes in lung function, reduced quality of life and functional status, and the onset of neuropsychiatric disorders up to 5 years after their critical illness. The cause, pathophysiological manifestation and clinical progress of patients with ARDS may be different from those of patients with other critical diseases. The hypothesis that ARDS has a distinct and unique pattern of long-term sequelae may derive from specific characteristics of ARDS: mechanical ventilation may be more harmful in ARDS than in other critical diseases; hypoxemia is a specific complication of ARDS, and survivors can face particular exposure to its negative effects; additionally, patients with ARDS receive specific therapies (extracorporeal membrane oxygenation, prone-position ventilation, muscle blockade and sedoanalgesia) that may influence the long-term outcome.^(49)^
